# In vitro anti-parasitic effect of the alkaloids harmaline and piperine on Toxoplasma gondii

**DOI:** 10.1590/S1984-29612024053

**Published:** 2024-09-16

**Authors:** Daniele Silva Souza Carreira, Carolina Emy Sato, Waléria Borges da Silva, Thereza Cristina Borio dos Santos Calmon de Bittencourt, Silvia Lima Costa, Rosângela Soares Uzêda

**Affiliations:** 1 Laboratório de Toxicologia e Fitoterapia, Hospital de Medicina Veterinária, Universidade Federal da Bahia – UFBA, Salvador, BA, Brasil; 2 Escola de Medicina Veterinária e Zootecnia, Universidade Federal da Bahia – UFBA, Salvador, BA, Brasil; 3 Departamento de Bioquímica e Biofísica, Universidade Federal da Bahia – UFBA, Salvador, BA, Brasil

**Keywords:** Toxoplasmosis, alkaloids, plant-derived substances, Toxoplasmose, alcaloides, substâncias derivadas de planta

## Abstract

*Toxoplasma gondii* is a coccidian protozoan of zoonotic importance that causes toxoplasmosis. Although the current treatments for toxoplasmosis may be associated with adverse effects and limited efficacy for different biological forms of the parasite, evidence suggests that alkaloid molecules such as harmaline and piperine exhibit antiparasitic effects against protozoa parasites. This investigation aimed to evaluate the *in vitro* effect of harmaline and piperine against *T*. *gondii* tachyzoites in infected Vero cell cultures. After 24 hours of host cell infection, the cultures were treated with harmaline or piperine (0.49 to 15.63 µg/mL). Negative and positive controls were RPMI/DMSO (0.1%) and sulfadiazine (200 µg/mL). Harmaline significantly reduced parasite multiplication by 20% compared to the negative control, while piperine decreased between 55.56% and 88.89% in a dose-dependent manner. According to an intracellular parasite proportion scale, it was observed that the Vero cells with low or moderate parasitic proliferation were more prevalent after the alkaloid treatment. The study demonstrated that the alkaloids had antiparasitic effects on *T*. *gondii*, with piperine being the most effective. Additional studies must be carried out to clarify other aspects of the action of the alkaloids on parasites.

## Resumo

*Toxoplasma gondii* é um protozoário coccídio de importância zoonótica, causador da toxoplasmose. Embora os tratamentos atuais para a toxoplasmose possam estar associados a efeitos adversos e à eficácia limitada para as diferentes formas biológicas do parasita, evidências sugerem que moléculas alcaloides, como a harmalina e a piperina exibem, efeitos antiparasitários contra protozoários parasitas. Essa investigação teve como objetivo avaliar o efeito *in vitro* da harmalina e da piperina contra taquizoítos de *T*. *gondii* em culturas de células Vero infectadas. Após 24 horas da infecção das células hospedeiras, as culturas foram tratadas com harmalina ou piperina (0,49 a 15,63 µg/mL). Os controles negativo e positivo foram RPMI/DMSO (0,1%) e sulfadiazina (200 µg/mL), respectivamente. Harmalina reduziu significativamente a multiplicação parasitária em 20% em comparação ao controle negativo, enquanto a piperina diminuiu entre 55,56% e 88,89%, de forma dose-dependente. De acordo com uma escala de proporção intracelular de parasitas, observou-se que células Vero com baixa ou moderada proliferação parasitária foram mais prevalentes após tratamento com alcaloides. O estudo demonstrou que os alcaloides tinham efeitos antiparasitários sobre o *T*. *gondii*, sendo a piperina mais eficaz. Estudos adicionais devem ser realizados para esclarecer outros aspectos da ação dos alcaloides sobre os parasitas.

## Introduction

*Toxoplasma gondii* is a protozoan parasite that has been identified as the causative agent of toxoplasmosis. The zoonotic potential of *T*. *gondii* is among the main reasons for taking measures against this parasite (Montoya & Liesenfeld, 2004).

Pyrimethamine and sulfadiazine are the current standard therapies for treating toxoplasmosis (Montoya & Liesenfeld, 2004). However, they have been linked with detrimental effects on patients and limited effectiveness against the various biological forms of the parasite throughout its life cycle (Al Nasr et al., 2016; Caumes et al., 1995; Luft & Remington, 1992).

It is well-known that plant-derived substances exhibit biological activity, including antiparasitic effects on *T. gondii* (Al Nasr et al., 2016). It is worth noting that secondary plant metabolites, which are distinct from the primary products of plant metabolism (such as sugars, proteins, nucleic acids, and chlorophyll, among others, that are essential for plant survival), have the potential to be used therapeutically (Harborne, 1999).

Secondary metabolites in plants exhibit a wide range of bioactive functions that inhibit competing plants, fungi, viruses, and bacteria (Wink, 2003). Additionally, these metabolites promote the self-regulation of plants during periods of water and salt stress and ultraviolet radiation (Isah, 2019; Yang et al., 2018).

Among secondary metabolites, alkaloids have drawn significant attention in pharmacology due to their potential benefits. Over 12,000 alkaloid molecules have been extensively studied and described (Wink, 2003).

The effects of alkaloids against *T. gondii* have already been reported, such as acridone and its derivatives (Holland Alday et al., 2021), colchicine (Aguirre-Cruz et al., 1996), harmina and its derivatives (Alomar et al., 2021), oxymatrine and matrine (Zhang et al., 2016), quinine (Fayer et al., 1972), as well as securinine and its derivatives (Holmes et al., 2011).

To contribute to the advancement of therapeutic options for toxoplasmosis, this work aimed to assess the antiparasitic effect of the alkaloids harmaline and piperine on *T. gondii* tachyzoites cultivated in Vero host cells.

## Materials and Methods

### Drugs and treatments

The alkaloids harmaline (214.26 g/mol, Product Number: 51330) and piperine (285.34 g/mol, Product Number: P49007) were acquired from Sigma-Aldrich Co. (Saint Louis, Missouri, USA) with purity levels above 90%. These alkaloids were then diluted in dimethyl sulfoxide (DMSO) to a stock concentration of 15 mg/mL and treatment concentrations as follows: 0.49, 0.98, 1.95, 3.91, 7.81, and 15.63 µg/mL.

Roswell Park Memorial Institute 1640 (RPMI) medium was used as the negative control with 0.1% DMSO, representing the maximum solvent concentration in alkaloid treatments. This control exhibits low cytotoxicity to host cells, as observed by Kim et al. (2002) and Silva et al. (2021). As the positive control, sulfadiazine was used (200 μg/mL in RPMI medium) commercialized by Drogavet® (Salvador, Bahia, Brazil).

### Vero cell and parasite strain cultures

The Vero cell line (ATCC CCL-81, Manassas, Virginia, USA) was cultivated in cell culture flasks (25 cm^2^) and maintained in RPMI medium with L-glutamine (Cytiva-Hyclone, Logan, Utah, USA), supplemented with 5% fetal bovine serum (FBS) (Invitrogen-Gibco, Grand Island, New York, USA) and 1% streptomycin, penicillin and amphotericin B (Invitrogen-Gibco, Grand Island, New York, USA) at 37°C with 5% CO_2_. The medium replacement was performed every 48 hours. Tachyzoites of *T*. *gondii* strain RH (ATCC 50174, Manassas, Virginia, USA) were cultivated in Vero cells and maintained in growth under the abovementioned conditions. The host cell-parasite culture was maintained until 60% of the destroyed area was reached, then expanded into subcultures.

### *In vitro* antiproliferative evaluation assay

The experimental procedure was conducted in 24-well plates, in which 13-mm circular slides were placed. Vero cells were dissociated from the monolayer using trypsin, followed by adding a culture medium. Subsequently, centrifugation was performed at 800 x *g* for 5 minutes at 4°C. The contents were then resuspended and counted in a Neubauer chamber to obtain a final concentration of 3x10 ^4^ cells/mL, adding 1 mL to each well. Vero cells were incubated for 24 hours at 37°C under 5% CO_2_.

The monolayer containing the culture of *T. gondii* was mechanically dissociated. Vero cells were disrupted to release tachyzoites with the 21G needle. Tachyzoites were purified on 5-µm filters (Minisart-Sartorius, Goettingen, DE). They were then centrifuged at 1,600 x *g* for 10 minutes at 4 ºC, followed by resuspension and counting in a Neubauer chamber, to obtain a final concentration of 1.5x10^2^ tachyzoites/µL (1.5x10^5^ tachyzoites/well), which were added to 24-well plates for cell Vero cell infection. This concentration maintains the ratio between host cells and tachyzoites at 5:1 (Sharif et al., 2019).

Infected plates returned to incubation for 24 hours. After that, controls and treatments were administered and maintained for 24 hours. Circular coverslips were removed from the wells, fixed with methanol, stained with Giemsa, and mounted on permanent slides that were evaluated through observation in an Olympus optical microscope magnified 1,000x and quantified intracellular tachyzoites for Vero cells.

### Statistical analysis

Statistical analysis was performed using the GraphPad Prism software (version 9.2.0).

Initially, whether the data followed a Gaussian distribution was checked using the Shapiro-Wilk and Kolmogorov-Smirnov tests with a p-value ≥ 0.05. Thus, hypothesis testing continued using Kruskal-Wallis non-parametric tests followed by Dunn's multiple comparisons test to analyze differences between concentrations of each alkaloid relative to controls. Correlations between the number of tachyzoites and alkaloid concentrations were assessed by calculating the Spearman correlation coefficient. The percentage of parasite reduction was calculated by using the number of counted tachyzoites and the following formula: Reduction (%) = ((Treatment - control)/control) x (-100). The Mann-Whitney test was used to check whether there were differences between alkaloids harmaline and piperine in each concentration. The frequency ranges were defined according to the maximum and minimum numbers of tachyzoites obtained from the counts, as well as from the proportion of inoculated parasites per cell, which was 5:1. Therefore, the proliferation scale was considered between 0 - 4 tachyzoites (low), 5 - 16 (moderate), 17 - 32 (high) and > 32 (very high). The chi-square test was also used to assess the proportions of tachyzoites per host cell. Median confidence intervals were established at a 95% confidence level, considering p-value < 0.05 as statistically significant.

## Results

### Antiproliferative activity of alkaloids on *T*. *gondii*

**Figure 1:**
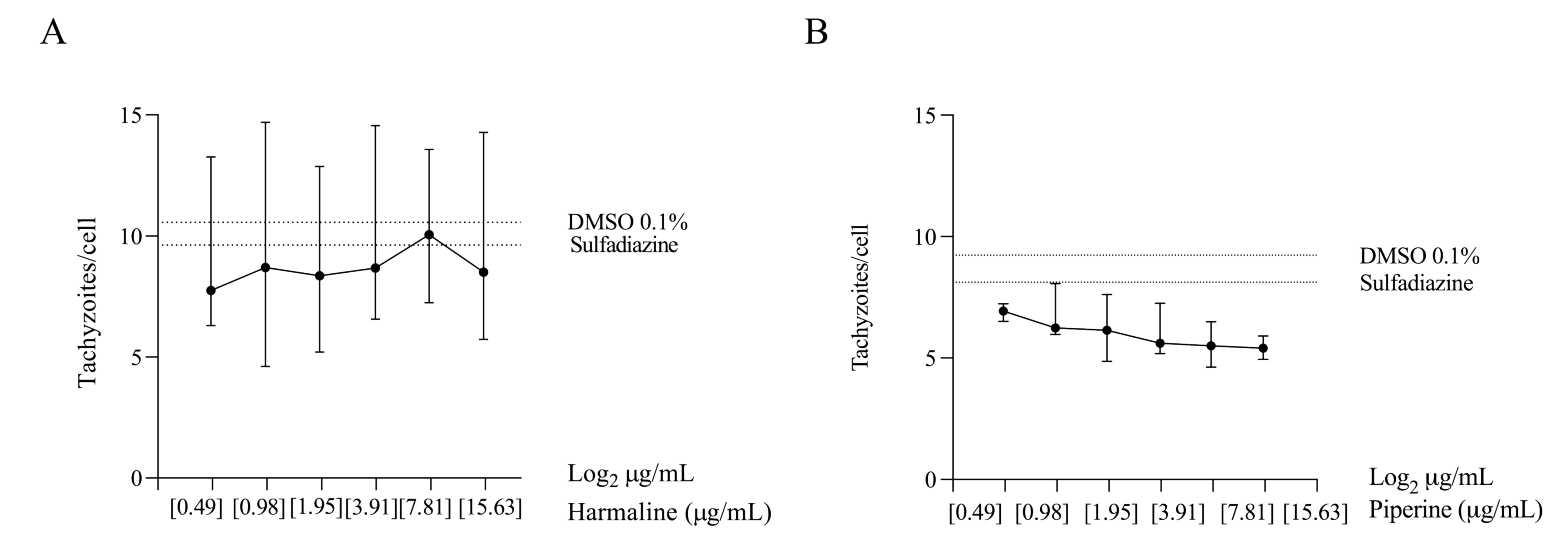
Number of *T. gondii* tachyzoites per Vero cells. (A) Harmaline; (B) Piperine. Data represent the median and interquartile range of the maximum count of 2,800 cells in two independent experiments.

In experiments carried out with *T*. *gondii*, it was observed that the response of cells treated with harmaline did not follow a dose-dependent profile within the evaluated concentration range, evidenced by Spearman's correlation analysis (Table 1). Besides that, except for the 7.81 µg/mL concentration, all evaluated doses presented median values ​​lower than those observed in the controls DMSO 0.1% and sulfadiazine (Figure 1A).

**Table 1:** Calculate Spearman’s correlation between each alkaloid concentration and the number of tachyzoites by cell. To consider the correlation of data, the level of significance considered p*<0.05*.

	Spearman’s *r*	Significance (*p*)
Harmaline	0.6000	0.2417
Piperine	-0.9429	0.0167

The treatment with piperine demonstrated a dose-dependent response in the concentration ranges evaluated, with a high correlation between data evidenced by Spearman's correlation analysis (Table 1). It is possible to observe that the median values ​​of the treatments are lower than the controls (Figure 1B).

**Figure 2:**
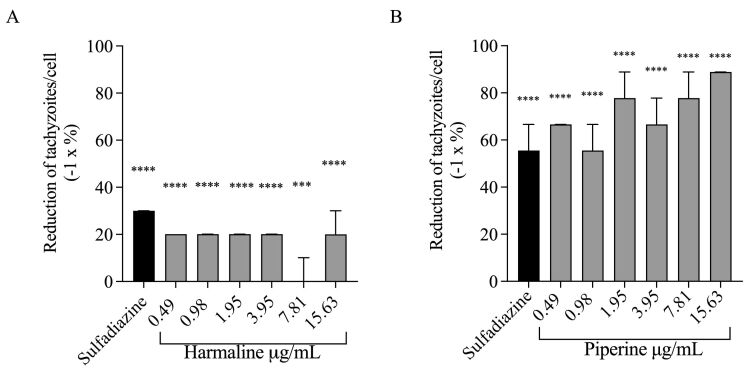
Percentage reduction of *T*. gondii tachyzoites per Vero cell. (A) Harmaline; (B) Piperine. The percentage was calculated in relation to the negative control DMSO 0.1%, expressed as median and 95% confidence interval. The Kruskal-Wallis test, followed by Dunn's multiple comparisons post-test, detected significant differences (*** p < 0.001; **** p < 0.0001).

Excepting for the concentration of 7.81 µg/mL, all other concentrations of harmaline reduced 20% of tachyzoites (Figure 2A). In the treatments with piperine reduction, intracellular tachyzoites were between 55.56% and 88.89% (Figure 2B).

**Figure 3:**
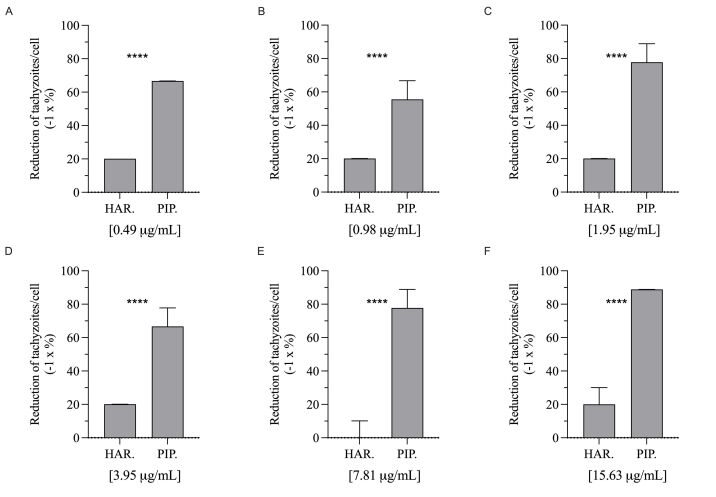
Comparison of the activity of harmaline (HAR) and piperine (PIP) against *T*. *gondii* (A-F). The data represent the comparison of the percentages of reduction of intracellular tachyzoites in each alkaloid at the same concentrations, expressed as median and 95% confidence interval. The Mann-Whitney test detected significant differences between alkaloids (**** p < 0.0001).

Comparing each concentration between two alkaloids, significant differences were observed between the reduction percentages of intracellular tachyzoites (Figure 3).

### Proportion of parasites in host cells

**Figure 4:**
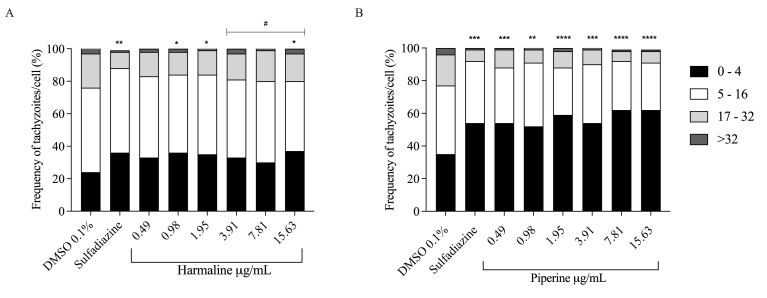
Relative frequency of *T. gondii* in treated Vero cells. (A) Harmaline; (B) Piperine. The data represent the maximum count of 2,800 cells from two independent experiments. Significant differences compared to 0.1% DMSO (* p < 0.05; ** p < 0.01; *** p < 0.001; **** p < 0.0001) and positive control (sulfadiazine) (# p < 0.05) were detected using the chi-square test.

When harmaline was used as a treatment (Figure 4A), it was noticed that about 30% to 37% of cells had 0-4 tachyzoites. In the range of 5-16 tachyzoites, the percentages varied from 43% to 50%. Additionally, tachyzoites between 17-32 were commonly found in about 14% to 19% of cells. Finally, in cells with more than 32 tachyzoites, the frequencies ranged from 2% to 3%.

The chi-square test revealed that the proportions of parasites in cells treated with harmaline were significantly different from the negative control at concentrations of 0.98, 1.95, and 15.63 µg/mL. However, concentrations of 3.91, 7.81, and 15.63 µg/mL exhibited differences compared to the positive control.

The cells treated with piperine (Figure 4B) exhibited a higher proportion of infected cells in the range corresponding to low parasite proliferation (0 – 4 tachyzoites), representing frequencies between 52% and 62%. Infection with 5 – 16 parasites was common in 29% to 39% of the cells. Other cells with 17 - 32 tachyzoites accounted for 6% to 11% of the cells. More than 32 parasites were observed in 1% to 2% of the cells.

Regarding the negative control, all the piperine treatments showed significant differences in proportions, but there were no differences compared to the positive control.

## Discussion

The biological activity of alkaloids harmaline and piperine in protozoa has been associated with *Trypanosoma* sp. (Freire-de-Lima et al., 2008; Ribeiro et al., 2004) and *Leishmania* sp. (Di Giorgio et al., 2004; Ferreira et al., 2011; Vieira-Araújo et al., 2018; Clemente et al., 2024). However, no reports were found regarding administering these alkaloids against *T*. *gondii****.***

In this study, when treating the Vero cells infected by *T*. *gondii* with harmaline or piperine was performed, a reduction in parasite proliferation was observed compared to the untreated control group.

*T*. *gondii* cultures did not respond to harmaline treatments in a dose-dependent manner. However, it was possible to observe parasite reductions of 20% at most concentrations. In a dose-dependent manner, Piperine showed more efficient results in parasite reduction than harmaline.

The dose of piperine 15.63 μg/mL (55 μM) in *T*. *gondii* showed a parasite reduction of 88.89%. In cultures of *L*. *amazonensis* promastigotes treated with 50 μM of piperine, a dosage very close to the maximum used in this work, the reduction corresponded to 53% in 24 hours of treatment. After 48 and 72 hours of treatment, it was possible to observe reductions of 92% and 96% respectively (Ferreira et al., 2011).

The alkaloid was also demonstrated to be effective against the amastigote stage of *L*. *amazonensis* in mouse macrophages; however, it was effective within 48 hours of treatment. Parasite counts were reduced by 63% (50 µM). However, for *L*. *braziliensis* amastigotes, Clemente et al. (2024) observed that the effective concentration for reducing parasites by 50% was equivalent to 8.76 µM after 48 hours of treatment, demonstrating greater efficiency for *L*. *braziliensis.*

When comparing the parasite reduction of harmaline and piperine at equivalent concentrations, treatments involving piperine exhibited a significantly greater decrease in parasite numbers than those of harmaline. This highlights the superior efficacy of piperine over harmaline in reducing the number of intracellular tachyzoites in this study.

The effectiveness of the treatment with harmaline or piperine was also evident when evaluating the parasite-cell relationship based on the proliferation scale established in the study. This scale considered the infection rate of the parasite-host cell (5:1), as well as the maximum and minimum values observed in the intracellular parasite count.

In the cultures treated with piperine, there was a higher occurrence of host cells with low parasitic proliferation (52 to 62%), and all the concentrations showed significant differences in relation to the untreated control. Although harmaline-treated cultures showed a higher frequency of cells with moderate parasite proliferation (43 to 50%), three concentrations had altered parasite-to-cell ratios compared to the negative control.

These results suggest that in terms of number of parasites per cell and their proportion, the treatments with alkaloids were different. Silva et al. (2021) reported that in a study carried out with gastrointestinal nematodes in goats, a remarkable ovicidal activity was observed in treatments with piperine and moderate activity with harmaline.

The results obtained in this study provide a significant contribution to the antiparasitic effects of harmaline since the literature is scarce on the subject, especially about protozoan parasites. So far, the effects of harmaline have only been reported by Di Giorgio et al. (2004), who described the substance as non-toxic to macrophages and active against *L*. *infantum*.

Many questions need to be addressed regarding the antiparasitic activity and the mechanism of action of alkaloids on parasites. However, harmaline and other β-carbonyl alkaloids have been associated with the ability to intercalate with DNA, which can disrupt the fidelity of DNA replication, leading to repair processes and other enzymatic and protein systems (Cao et al., 2007).

The antiparasitic activity of piperine in organisms such as *L*. *infantum* (Ferreira et al., 2011) and *Trypanosoma cruzi* (Freire-de-lima et al., 2008) was associated with the entrapment of parasites in the G1 and S phase of the cell cycle, interruption of parasite cytokinesis, interference with the synthesis of essential lipids and ultrastructural damage.

As it was already observed *in silico* that the piperine presented superior affinity values for the enzyme *Plasmodium falciparum* enoyl-ACP reductase (P*f*ENR) when compared to the reference inhibitor triclosan, this enzyme has a determinant role in the fatty acid biosynthesis of *Plasmodium falciparum* and is absent in humans (Silva et al., 2020). This suggests that the decrease in the proliferation rate of *T*. *gondii* may also be related to the cell cycle.

Low toxicity characteristic in host cells is a crucial aspect to consider in the search for new therapeutic compounds with antiparasitic properties, as the objective is to reduce or eliminate parasitic forms without harming the host. In a previous study conducted by our group (Silva et al., 2021), the cytotoxicity of piperine and harmaline on Vero cells was evaluated through MTT colorimetric assays. The dosage ranges used in this investigation were deliberately chosen based on their minimal toxicity to these specific host cells.

The alkaloids herein evaluated showed an antiparasitic effect at doses considered to be of low toxicity to host cells. Harmaline and piperine can be considered as potential therapeutic drugs for toxoplasmosis, and piperine was more effective than harmaline. However, one still needs to elucidate the mechanisms of antiparasitic action and carry out clinical trials to validate the substances in compositions of therapeutic protocols.
